# Mitoxyperilysis: Rethinking oxidative stress as a spatially constrained lethal signal

**DOI:** 10.7150/ijbs.130535

**Published:** 2026-03-30

**Authors:** Chen-Yueh Wen, Meng-Yu Wu, Andy P. Tsai, Su-Boon Yong, Chia-Jung Li

**Affiliations:** 1 Division of Urology, Show Chwan Memorial Hospital, Changhua 500, Taiwan.; 2 Division of Urology, Chang Bing Show Chwan Memorial Hospital, Changhua 500, Taiwan.; 3 Department of Emergency Medicine, Taipei Tzu Chi Hospital, Buddhist Tzu Chi Medical Foundation, New Taipei 231, Taiwan.; 4 School of Medicine, Tzu Chi University, Hualien 970, Taiwan.; 5 Department of Neurology and Neurological Sciences, Stanford University School of Medicine, Stanford, CA 94305, USA.; 6 Department of Allergy and Immunology, China Medical University Children's Hospital, Taichung 404, Taiwan.; 7 Research Center for Allergy, Immunology, and Microbiome (A.I.M.), China Medical University Hospital, Taichung 404, Taiwan.; 8 Department of Obstetrics and Gynecology, Kaohsiung Veterans General Hospital, Kaohsiung 813, Taiwan.; 9 Institute of Biopharmaceutical Sciences, National Sun Yat-sen University, Kaohsiung 804, Taiwan.; 10 National Museum of Marine Biology & Aquarium, Pingtung 944, Taiwan.

A longstanding frustration in regulated cell death research is that oxidative stress often predicts pathology yet fails to yield consistently effective therapies when approached as a bulk biochemical variable. A recent *Cell* study proposes a provocative solution to this mismatch by defining a lytic, inflammatory death program in which oxidative injury becomes lethal only after it is spatially “licensed” at the plasma membrane, a two-stage process termed mitoxyperiosis followed by mitoxyperilysis 1. Here, 'spatial licensing' is defined as the requisite physical anchoring of damaged mitochondria at the plasma membrane, which concentrates oxidative injury within a vulnerable microdomain to surpass local repair capacities, a process distinct from bulk biochemical stress. In this framework, immune activation and metabolic disruption jointly generate a pool of oxidatively damaged mitochondria, but cell rupture is dictated by whether those mitochondria are retained long enough at the cell periphery to deliver short lived reactive species into a vulnerable membrane microdomain. The conceptual shift is subtle but consequential for biomedicine: the decisive variable is not the global abundance of reactive oxygen species, but the subcellular topology that concentrates damage where repair and buffering are locally outcompeted. Specifically, 'spatial licensing' denotes a mechanism where the lethal outcome of oxidative injury is contingent upon the precise subcellular positioning and physical anchoring of organelles, rather than bulk biochemical variables. Mechanistically, mitoxyperiosis refers to the spatially localized delivery of reactive species leading to membrane-proximal lipid peroxidation, whereas mitoxyperilysis denotes the eventual physical rupture of the plasma membrane and inflammatory cell death.

For clarity, the mitoxyperilysis framework comprises two sequential and mechanistically distinct steps. Mitoxyperiosis refers to the initial phase in which oxidatively damaged mitochondria are repositioned and retained at the plasma membrane, enabling spatially confined delivery of reactive oxygen species and membrane-proximal lipid peroxidation. Mitoxyperilysis, in contrast, denotes the downstream consequence of this spatial licensing process, culminating in irreversible plasma membrane rupture and inflammatory lytic cell death. Reiterating this two-step model is essential for distinguishing localized oxidative priming from the terminal lytic outcome, particularly for readers newly encountering this conceptual framework. This “where not just what” logic resonates with the broader organelle biology revolution driven by membrane contact sites 2. Contact sites are now recognized as hubs that integrate signaling, lipid metabolism, and stress responses, often through highly localized biochemical microenvironments that cannot be inferred from whole cell measurements 2. Importantly, mitochondria can form specialized interactions with the plasma membrane in multiple contexts, and this axis has been argued to be underappreciated relative to classical mitochondria endoplasmic reticulum contacts 3. Mitoxyperilysis can be read as a death program that exploits these proximity principles, turning mitochondria plasma membrane adjacency into an execution platform for membrane proximal lipid peroxidation and rupture 1, 3. For *Int J Biol Sci* readers, this offers a useful interpretive lens: many disease models that are framed as “oxidative stress driven” may actually be governed by spatially constrained oxidative lesions that emerge only under specific trafficking, tethering, or cytoskeletal states 2.

The *Cell* study identifies mTORC2 as a central switch that governs whether oxidative injury remains broadly tolerated or becomes spatially focused and lytic 1. This is biologically plausible because mTORC2 has long been connected to actin cytoskeletal regulation and cell surface dynamics, processes that can control organelle positioning and contact persistence. The implication for pharmacology is that mTORC2 and downstream cytoskeletal circuitry function as key spatial regulators that modulate the threshold for lytic failure by tuning the duration and geometry of mitochondria-plasma membrane contact 4. In practical terms, inhibiting oxidant production might be insufficient if mitochondria remain trapped at the membrane, while shortening contact duration might preserve membrane integrity even in the presence of substantial mitochondrial oxidative stress 1.

This idea has immediate relevance to the 2025 original research portfolio of *Int J Biol Sci*, which has repeatedly highlighted oxidative stress coupled to regulated necrosis as a pharmacologically tractable node in organ injury. In acute kidney injury, Wei and colleagues show that remote ischemic preconditioning protects renal function and mitigates tubular epithelial ferroptosis, with mechanistic evidence pointing to suppression of NOX4 driven ROS signaling and preservation of mitochondrial homeostasis 5. While ferroptosis is typically conceptualized as a lipid peroxidation driven death state, mitoxyperilysis suggests an added layer: whether lipid peroxidation is diffusely distributed or concentrated at plasma membrane adjacent microdomains could influence whether cells progress to frank membrane rupture and inflammatory lysis 1, 5. This provides a theoretical basis for testable predictions in the kidney setting preconditioning might protect not only by lowering oxidative burden, but also by preventing spatial retention of damaged mitochondria at the cell cortex, thereby reducing the probability of topology licensed lysis during inflammatory metabolic stress 1, 3, 5.

A parallel opportunity emerges in myocardial ischemia reperfusion injury, where Xu and colleagues report that activation of sphingosine 1 phosphate receptors alleviates cardiomyocyte injury by mitigating oxidative stress and suppressing ferroptosis, including via transcriptional upregulation of antioxidant and anti ferroptotic genes and improved mitochondrial integrity 6. Here again, mitoxyperilysis offers a potential framework for reframing therapeutic evaluation drugs that shift antioxidant capacity may not equivalently prevent cell rupture if mitochondria are spatially positioned to deliver damage directly to the sarcolemma 1, 6. A compelling translational extension is that cardioprotective agents such as sphingolipid pathway modulators could be benchmarked not only by conventional ROS and lipid peroxidation markers, but also by spatial pharmacodynamic endpoints including mitochondria sarcolemma contact duration and membrane proximal lipid oxidation in stressed cardiomyocytes 1, 6. Such endpoints may help discriminate interventions that truly prevent lytic failure of the plasma membrane from those that merely dampen global redox signals 1.

The mitoxyperilysis concept also intersects with *Int J Biol Sci* work on inflammatory lytic death pathways beyond ferroptosis. Yu and colleagues show that exogenous spermidine protects against trastuzumab mediated cardiomyocyte injury and pyroptosis through SIRT3 regulated mitochondrial quality control, reducing mitochondrial oxidative stress and preserving mitochondrial function 7. Pyroptosis is canonically executed by gasdermin mediated pore formation that culminates in membrane rupture and release of inflammatory mediators 8. Mitoxyperilysis does not rely on gasdermin pores, but it converges on an outcome that matters clinically, namely inflammatory lysis 1, 8. This invites a synthesis: in cardiotoxicity contexts where mitochondria are damaged and inflammatory signaling is present, multiple lytic “routes” may coexist, including pore mediated rupture and topology licensed oxidative rupture 1, 7, 8. Therapeutic strategies that bolster mitochondrial quality control, such as SIRT3 dependent programs, may therefore blunt more than one lytic axis by reducing the pool of mitochondria capable of driving membrane proximal oxidative lesions while also attenuating upstream inflammatory amplification.

A further advantage of situating mitoxyperilysis alongside ferroptosis and pyroptosis is that both of these established programs have already produced tractable chemical biology tools and translational hypotheses. Ferroptosis was defined as an iron dependent, lipid ROS driven non apoptotic death process and has since become a major target in cancer, neurodegeneration, and ischemic injury 9. Pyroptosis has likewise matured into a drug discovery arena focused on inflammasomes, inflammatory caspases, and gasdermin pore formation 8. Mitoxyperilysis can be viewed as orthogonal to these pathways yet potentially overlapping at the level of membrane vulnerability: lipid peroxidation, membrane repair capacity, and the spatial delivery of reactive species likely modulate whether cells undergo sublethal stress, regulated demise, or catastrophic rupture 1, 8, 9(Table 1). Rather than functioning as a mere modulatory layer, mitoxyperilysis represents a standalone lytic program that is defined by its absolute requirement for subcellular organelle positioning. Although it may converge with ferroptosis during the final stage of lipid-driven membrane failure, the upstream spatial gate, which involves the recruitment and retention of mitochondria at the plasma membrane, serves as a distinct regulatory checkpoint that is not present in classical ferroptotic or pyroptotic models. This is where* Int J Biol Sci* can play an outsized role: by encouraging studies that integrate classic biochemical markers with spatially resolved readouts, the journal can help define when a therapy prevents regulated death versus merely delaying the moment of lysis 1, 2.

What, then, is the actionable roadmap for a field that has historically measured oxidative stress in bulk? The first priority is assay evolution. At the same time, it is important to acknowledge current experimental and technical limitations associated with measuring spatial pharmacodynamic endpoints. Quantifying mitochondria-plasma membrane contact duration, contact stability, and membrane-proximal lipid peroxidation typically requires advanced live-cell imaging, high-resolution spatial probes, and precise temporal alignment between organelle positioning and membrane rupture. These approaches remain technically demanding and are not yet standardized across model systems. Accordingly, while spatially resolved readouts hold substantial promise for improving therapeutic discrimination, their implementation currently represents an evolving methodological frontier rather than a routine pharmacological endpoint. The *Cell* work implies that pharmacological screens should include imaging based or proximity-based metrics that quantify mitochondria plasma membrane contact persistence, membrane proximal lipid peroxidation, and the temporal order linking contact formation to rupture. The second priority is mechanistic expansion: mitochondria plasma membrane interactions are incompletely mapped in mammalian cells, and defining the molecular tethers, motors, and actin remodeling nodes that stabilize subplasmalemmal mitochondria will expand druggable space 3. The third priority is context specificity: because mitoxyperilysis is triggered by synergy between innate immune activation and metabolic disruption, models that combine inflammatory cues with nutrient limitation or mitochondrial stress may reveal therapeutic liabilities that are invisible in single stress systems. Finally, the therapeutic logic is dual: in inflammatory organ injury, one might suppress spatial licensing to preserve barrier integrity, whereas in cancer, one might exploit topology control to force lytic collapse selectively in immunometabolically stressed tumors 1.

In sum, mitoxyperilysis reframes oxidative stress as a spatially gated problem whose decisive step is organelle positioning at the plasma membrane 1. The 2025* Int J Biol Sci* original articles on renal ferroptosis modulation by remote ischemic preconditioning, cardiomyocyte ferroptosis suppression by sphingosine 1 phosphate receptor activation, and cardiomyocyte pyroptosis mitigation by spermidine driven mitochondrial quality control all converge on a shared principle: mitochondrial integrity and redox handling are necessary but may be insufficient descriptors of lytic fate without considering subcellular topology 5-7. By prioritizing spatial pharmacology endpoints and mechanistic work on mitochondria plasma membrane interfaces, *Int J Biol Sci* can help transform a new death concept into a predictive and therapeutically actionable framework spanning organ injury, cardiometabolic disease, and cancer biology 1-3.

## Figures and Tables

**Figure 1:**
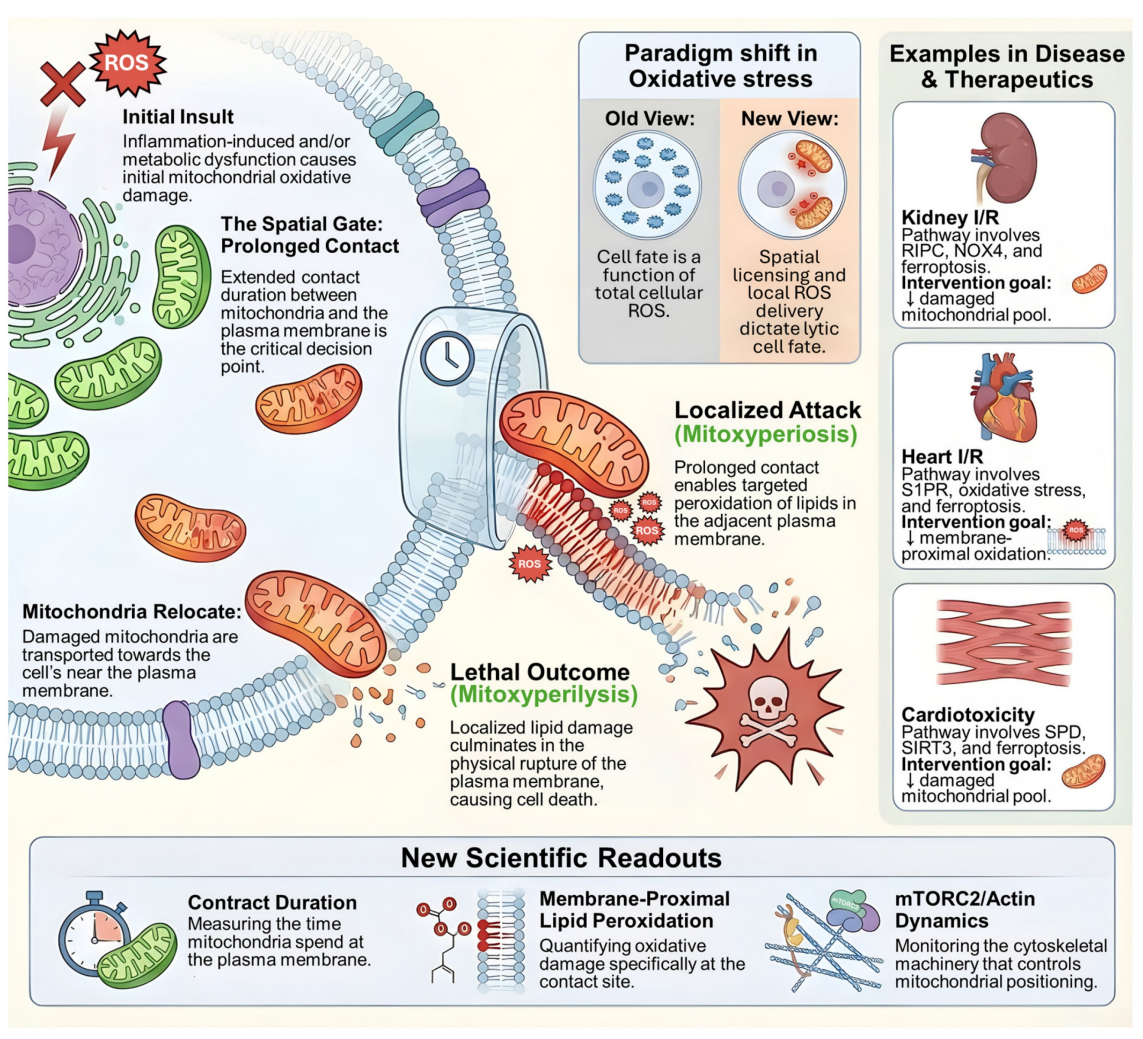
** Mitoxyperilysis as a spatially licensed inflammatory lytic cell death pathway.** Schematic overview of mitoxyperilysis, a newly identified form of lytic cell death driven by spatially constrained mitochondrial oxidative damage. Inflammatory and metabolic stress induces mitochondrial injury and relocation of damaged mitochondria toward the plasma membrane. Prolonged mitochondria-plasma membrane contact acts as a spatial gate that enables localized delivery of reactive oxygen species, leading to membrane-proximal lipid peroxidation (mitoxyperiosis). Accumulation of localized lipid damage ultimately results in plasma membrane rupture and inflammatory lytic cell death (mitoxyperilysis). The figure illustrates a paradigm shift from global oxidative damage to spatially licensed injury as the determinant of lethality. Disease contexts including kidney ischemia-reperfusion injury, myocardial ischemia-reperfusion injury, and cardiotoxicity are shown, together with key translational readouts such as mitochondria-membrane contact duration, membrane-proximal lipid peroxidation, and mTORC2/actin-dependent cytoskeletal dynamics.

**Table 1:** Comparative features of regulated lytic cell death pathways.

Feature	Mitoxyperilysis	Ferroptosis	Pyroptosis
Primary triggers	Innate immune activation + Metabolic disruption	Iron accumulation + GPX4 inhibition	Inflammasome activation + Pro-inflammatory caspases
Key executor	Localized ROS delivery at MPM contact sites	Generalized lipid peroxidation	Gasdermin pore formation
Spatial licensing	Required (Mitochondria-PM adjacency)	Not strictly required (Diffuse)	Not strictly required (Diffuse pores)
Pharmacological target	mTORC2 / Cytoskeletal tethers	Iron chelators / Liproxstatin-1	NLRP3 inhibitors / Gasdermin blockers
Biomarkers	MPM contact duration; Membrane-proximal lipid oxidation	Iron levels; Bulk C11-BODIPY staining	Cleaved Gasdermin D; IL-1β/IL-18 release
